# Gut Microbiota Differences in Down Syndrome Are Most Pronounced in Childhood and Diminish With Age

**DOI:** 10.1155/ijm/6617119

**Published:** 2026-07-09

**Authors:** Jesús M. Pérez-Villarreal, Wendy Gastélum Espinoza, Kenia Esparza Ocampo, Alberto K. De la Herrán Arita, Alma Guadrón Llanos, Carla Angulo Rojo, Loranda Calderón Zamora, Claudia Norzagaray Valenzuela, Yair Cruz-Narváez, Jaime García-Mena, Javier Magaña Gómez

**Affiliations:** ^1^ Posgrado en Ciencias Biomédicas, Facultad de Ciencias Químico-Biológicas, Universidad Autónoma de Sinaloa, Culiacán, Sinaloa, Mexico, uas.edu.mx; ^2^ Facultad de Medicina, Universidad Autónoma de Sinaloa, Culiacán, Sinaloa, Mexico, uas.edu.mx; ^3^ Programa de Nutrición, Universidad Autónoma de Occidente, Culiacán, Sinaloa, Mexico, uao.edu.co; ^4^ Posgrado en Ciencias Biológicas, Facultad de Biología, Universidad Autónoma de Sinaloa, Culiacán, Sinaloa, Mexico, uas.edu.mx; ^5^ Escuela Superior de Ingeniería Química e Industrias Extractivas, Instituto Politécnico Nacional, Ciudad de México, Mexico, ipn.mx; ^6^ Departamento de Genética y Biología Molecular, Centro de Investigación y de Estudios Avanzados del Instituto Politécnico Nacional (Cinvestav), Ciudad de México, Mexico; ^7^ Facultad de Ciencias de la Nutrición y Gastronomía, Universidad Autónoma de Sinaloa, Culiacán, Sinaloa, Mexico, uas.edu.mx

**Keywords:** Down syndrome, early-life interventions, gut microbiota, metabolic, microbiota dysbiosis, neurodegenerative disease, V4-16S rRNA sequencing

## Abstract

**Introduction:**

Down syndrome (DS) is linked to increased risks of metabolic, gastrointestinal, and neurodegenerative disorders. Early alterations in the gut microbiota have potential long‐term health impacts. This report appears to be the first study to stratify DS participants by age to explore early‐life microbiota changes.

**Methodology:**

We conducted a cross‐sectional analysis of gut microbiota in children, adolescents, and adults with DS, compared with a control group, using Illumina iSeq100 sequencing of the V4 polymorphic region of the 16S rRNA gene.

**Results:**

Children with DS exhibited lower microbial diversity and a higher abundance of genera such as *Sutterella* and *Enterococcus*. These differences lessened in older groups, indicating a convergence with control profiles.

**Conclusion:**

Early alterations in gut microbiota in DS may contribute to metabolic and neurodegenerative risks, emphasizing the need for early interventions to potentially improve long‐term health outcomes.

## 1. Introduction

Down syndrome (DS) is the most common genetic condition worldwide. As life expectancy increases in this population, individuals with DS face a higher risk of developing metabolic disorders (e.g., obesity and Type 2 diabetes), gastrointestinal issues such as chronic constipation, mood disorders including anxiety and depression, and neurodegenerative diseases like Alzheimer′s disease [1–6]. Moreover, DS is linked to immune dysregulation, characterized by elevated proinflammatory markers, which can lead to reduced quality of life and increased risk of chronic metabolic and neurodegenerative conditions [7–9].

The gut microbiota maintains host health by producing short‐chain fatty acids (SCFAs), some neurotransmitters, and other metabolites involved in energy homeostasis, immune modulation, and brain development. Several factors, including mode of delivery, diet, antibiotic exposure, and host genetics, influence its composition [10]. Alterations in the gut microbiota, particularly during early developmental windows, have been linked to long‐term health outcomes like obesity, insulin resistance, neuroinflammation, mood disorders, and neurodegenerative diseases [11]. Emerging research has shown that assisted modification of gut microbiota during critical developmental periods may offer age‐specific therapeutic benefits [12], emphasizing the importance of maintaining a healthy microbiota early in life.

Despite these findings, only a few studies have explored the gut microbiota profile in individuals with DS. Preliminary reports suggest that specific bacterial genera, such as *Sutterella*, *Blautia*, and *Citrobacter*, may be associated with behavioral and cognitive impairments in both children and adults with DS [13, 14]. More recently, it has been demonstrated in a murine model that fecal microbiota transplantation from people with DS, especially those with a high abundance of *Prevotella*, can induce proinflammatory cytokine production and behavioral alterations suggesting a causal role of gut dysbiosis in neurodevelopmental and behavioral changes [7].

Taken together, these findings support the hypothesis that a proinflammatory gut microbiota may compromise the integrity of the gut and blood–brain barriers, thereby impairing systemic and cerebral metabolism and promoting the onset of obesity, diabetes, behavioral disorders, and Alzheimer′s disease in the DS population [15–21]. Therefore, the present study is aimed at characterizing the gut microbiota composition across different age groups in individuals with DS, from childhood to adulthood. Notably, this is the first study to stratify DS participants by age, highlighting the relevance of early‐life alterations in the microbiota and their potential long‐term consequences [22–24].

## 2. Materials and Methods

### 2.1. Study Design and Subject Selection

An observational, cross‐sectional, and comparative study was conducted involving individuals with and without DS. Participants were recruited by open invitations at multidisciplinary health fairs. All participants with DS were accompanied by a parent or legal guardian. Written informed consent was obtained from the legal guardians of all participants. For adult participants with cognitive impairment, consent procedures were in accordance with ethical guidelines, and assent was obtained whenever possible. The study was approved by the Research Ethics Committee of the Faculty of Medicine, Autonomous University of Sinaloa (Approval Number: 175123), and conducted in accordance with the Declaration of Helsinki. The diagnosis of DS was confirmed by karyotype analysis of cultured blood cells using Roswell Park Memorial Institute (RPMI) 1640. The cell cycle was arrested with colchicine, and chromosome staining was performed with Giemsa and trypsin for subsequent microscopic observation. Exclusion criteria included a history of using nutritional supplements, special diets, or consuming antibiotics or probiotic supplements within 6 months before sample collection. The final cohort consisted of 34 individuals with DS and 38 age‐ and sex‐matched controls, subdivided into children (4–12 years) and adolescents–adults (> 12 years): 27 control children, 18 children with DS, 11 control adolescents–adults, and 16 adolescents–adults with DS (Figure S1).

### 2.2. Clinical Data and Specimen Collection

A clinical history was obtained from each participant. Anthropometric measurements were performed following the International Society for the Advancement of Kinanthropometry (ISAK) standardized procedures. Body weight was measured using an electronic scale (Tanita HS‐302, United States) with participants wearing light clothing, and height was measured with a stadiometer (SECA 213, United States). Body mass index (BMI) was calculated (kg/m^2^), and individuals were classified according to the BMI‐for‐age percentiles from the National Institute of Pediatrics reference tables for the Mexican population [25].

Fasting blood samples were collected by venipuncture after a 12‐h fast and subsequently centrifuged at 3000 × g for 20 min to obtain serum. The serum was transported to the Center for Research and Teaching in Health Sciences for evaluation of biochemical parameters and thyroid hormone profiles. Fecal samples were collected in sterile containers and preserved at −70°C until further processing.

### 2.3. DNA Extraction

Using the ZymoBIOMICS DNA Miniprep kit (Cat. D4300, Zymo Research) according to the manufacturer′s instructions, 200 mg of a fresh stool sample was used for DNA extraction using bead lysis, followed by purification of the genetic material using columns and final elution using RNase‐free water. The integrity of the extracted DNA was verified by electrophoresis on a 0.5% agarose gel (90 V, 50 min), and purity was assessed using absorbance ratios (A260/280 and A260/230) measured with a NanoDrop 2000 spectrophotometer (Thermo Fisher Scientific, Massachusetts, United States).

### 2.4. Amplification of the V4 Region of the Bacterial 16S rRNA Gene

The V4 region of the bacterial 16S rRNA gene was amplified by endpoint PCR using forward in house designed primer iSeq V4‐520F (5 ^′^TCG TCG GCA GCG TCA GAT GTG TAT AAG AGA CAG‐*AYT GGG YDT AAA GNG*‐3 ^′^), where the first 33 bases were taken from the “16S amplicon PCR forward primer” [26] and the last 15 italicized bases complementary to coordinates 562‐576 of the *Escherichia coli* 16S rDNA molecule *rrnB* GenBank J01859.1 [27] were taken from a systematic study of microbial diversity investigations using 16S rRNA amplicon genes [28]. The reverse primer was 16S amplicon PCR reverse primer 5 ^′^‐GTC TCG TGG GCT CGG AGA TGT GTA TAA GAG ACA G‐*GAC TAC HVG GGT ATC TAA TCC*‐3 ^′^, where the last 15 italicized bases are complementary to coordinates 784‐805 of the *E. coli* 16S rDNA molecule *rrnB* GenBank J01859.1 [27]. These primers generated a 311‐bp amplicon [26]. PCR reactions were performed using Phusion High‐Fidelity DNA Polymerase (Thermo Fisher Scientific) in a total volume of 25 *μ*L. The thermal cycling conditions consisted of an initial denaturation at 95°C for 3 min, followed by 25 cycles of denaturation at 95°C for 30 s, annealing at 55°C for 30 s, and extension at 72°C for 30 s. A final extension step at 72°C for 3 min was included. PCR products were purified with Agencourt AMPure magnetic purification beads (Beckman Coulter, Brea, California, United States). A second PCR was performed to add indexes using the Illumina Nextera XT Index Kit v2 Set D (96 indexes, 192 samples) for multiplexing. Following amplification, a second purification step was performed. Library quantification was carried out using the Qubit 2.0 fluorometer (Thermo Fisher Scientific, Invitrogen, Q32866). The library size distribution was verified using the Agilent 2100 Bioanalyzer system and the Agilent High Sensitivity DNA kit (No. 6067‐4626), yielding a final fragment size of 388 bp. Libraries were normalized to a final concentration of 1 nM. PhiX was included as internal quality control. Sequencing was performed on the Illumina iSeq 100 platform with a paired‐end 2 × 150 − bp configuration. The sequence FASTQ R1 files and corresponding mapping files for all samples used in this study were deposited in the NCBI repository BioProject: PRJNA1284013 (https://www.ncbi.nlm.nih.gov/bioproject/PRJNA1284013).

### 2.5. Statistical and Bioinformatics Analysis

Descriptive data are presented as mean ± standard deviation (SD) and were analyzed using Prism 8.0 software (GraphPad Software, San Diego, California, United States). Depending on data distribution, comparisons between groups were performed using the Student′s *t*‐test or the Mann–Whitney *U*‐test. Amplified sequence variants (ASVs) were inferred from high‐quality single‐end R1 reads (151 bp, > Q30) using the DADA2 pipeline in QIIME2‐2024.2, which handles denoising, chimera removal, and ASV table/representative sequence generation in a single step (rep‐seqs‐dada.qza/qzv: 1956 sequences). Following DADA2 processing, 11,313 reads per sample were retained for subsequent analyses. Although initial paired‐end sequencing (150 × 2 bp) produced 242‐bp merged fragments via PEAR, QIIME2 DADA2 processing yielded few representative sequences, so data were reprocessed as single‐end R1 reads for all final ASV‐based analyses (no OTUs used). Taxonomic assignment used qiime feature‐classifier classify‐consensus‐blast at 97% similarity against SILVA 138.2‐ssu‐nr99.seqs.qza/138.2‐ssu‐nr99.tax.qza. All QIIME2 artifacts, workflows, raw data, and R scripts are available at https://github.com/mcjesusmpv.

Downstream analyses were performed in R 4.2.1 via RStudio. Data were imported using the qiime2R (Version 0.99.6) package, and microbial community analyses, including relative abundances, were conducted using the phyloseq (Version 1.40.0) package. Core microbiota (prevalence, ≥ 20%; detection threshold, ≥ 1%) were visualized using the microbiome (Version 1.18.0) and ComplexHeatmap (Version 2.12.1) packages. Spearman correlation analyses were conducted after filtering ASVs to ≥ 25% prevalence and applying a log1p transformation, and anthropometric and biochemical variables were standardized using *Z*‐scores. Rarefaction curves were generated to assess sequencing depth and sample diversity. Alpha diversity metrics were calculated, including Shannon, Simpson, and Chao1. Given the modest and unbalanced size of the subgroups, we performed effect size analyses (Wilcoxon *r* and *η* ^2^) to assess the biological relevance of the observed differences. Beta diversity was evaluated using weighted and unweighted UniFrac distance and visualized through nonmetric multidimensional scaling (NMDS). Differential abundance analysis was performed using DESeq2 with the Model Design = ~Group + Age. Age was included as a covariate due to its recognized influence on gut microbiota composition and availability for all participants; BMI, although available, was excluded from the primary model to prevent overparameterization and power loss given the sample size. Sequencing depth normalization was performed via DESeq2′s internal size factor estimation, with complete output tables (log2 fold changes, raw *p* values, and false discovery rate [FDR]–adjusted *p* values) now in the Supporting Information (DESeq2_results.xlsx). A confirmatory ANCOM‐BC analysis addressed compositional data concerns and small‐sample sensitivity.

Functional profiling of the bacterial metagenome was predicted using PICRUSt2 v2.6.2 with the GTDB reference database. To enhance prediction reliability, ASVs with NSTI values > 2 were excluded from downstream analyses. KEGG Orthologs obtained from PICRUSt2 were then used for pathway enrichment analysis based on the KEGG database. Mean NSTI values per sample and group distributions are now provided in Supporting Information “Weighted_NSTI_Picrust2.xlsx.” Full KEGG Orthology (KO) tables and pathway enrichments are available in “KEGG_enrichment.xlsx.” Statistical analyses were performed using Fisher′s exact test, with multiple testing corrected using the Benjamini–Hochberg (BH) method. The FDR was calculated using the BH method to control for multiple comparisons. A *p* value of < 0.05 was considered statistically significant. All artifacts, files, Qiime2 workflow, raw data, and Rscripts were uploaded to GitHub (https://github.com/mcjesusmpv).

## 3. Results

### 3.1. Participant Characteristics

Anthropometric and biochemical characteristics are shown in Table 1. Children with DS had significantly lower heights than those in the control group. Concerning biochemical parameters, individuals with DS, especially in the children group, showed lower levels of high‐density lipoprotein cholesterol (HDL‐c). Importantly, this difference in HDL‐c levels remained even after adjustment for BMI, suggesting it was independent of weight status. No significant differences were found for other biochemical variables between the adolescents and adult groups.

**Table 1:** Anthropometric and biochemical characteristics of study groups.

Variables	Control Children *N* = 27^a^	DS/children *N* = 18^a^	*p*	Control adolescents–adults *N* = 11^a^	DS/adolescents–adults *N* = 16^a^	*p*
Age (years)	9.2 ± 1.9	8.2 ± 2.6	0.19	25.1 ± 12.1	22.5 ± 9.7	0.65
Height (m)	1.4 ± 0.1	1.2 ± 0.2	0.01 ^∗^	1.6 ± 0.1	1.5 ± 0.1	0.01 ^∗^
Weight (kg)	33.6 ± 10.1	28.5 ± 11.6	0.16	58.2 ± 14.8	57.1 ± 15.7	0.98
Glucose (mg/dL)	97.6 ± 18.4	95.1 ± 7.9	0.65	90.2 ± 13.1	92.7 ± 5.7	0.32
Triglycerides (mg/dL)	80.7 ± 35.4	91.1 ± 32.1	0.23	90.4 ± 58.9	99.6 ± 41.8	0.29
Cholesterol (mg/dL)	170.3 ± 37.5	159 ± 36.7	0.41	185.1 ± 46.9	162.1 ± 39.2	0.18
HDL‐c (mg/dL)	61.8 ± 17.9	45.2 ± 11.3	0.01 ^∗^	57.7 ± 26.3	47.7 ± 12.3	0.13
LDL‐c (mg/dL)	92.3 ± 23.2	95 ± 31.1	0.89	109.3 ± 30	95.2 ± 34.8	0.16
VLDL‐c (mg/dL)	16.1 ± 7.1	18.2 ± 6.4	0.19	18.1 ± 11.7	19.9 ± 8.4	0.29

*Note:* The Mann‐Whitney *U* test was used to identify statistical differences (denoted by ∗; *p* < 0.05).

Abbreviations: BMI, body mass index; HDL‐c, high‐density lipoprotein cholesterol; LDL‐c, low‐density lipoprotein cholesterol; VLDL‐c, very low‐density lipoprotein cholesterol.

^a^Mean ± SD.

### 3.2. Relative Abundance and Core Microbiota

Analysis of microbiota composition at the phylum level in children with and without DS revealed that Bacillota was the most abundant, followed by Bacteroidota, Actinomycetota, Pseudomonadota, and Verrucomicrobiota. Statistically significant differences included a higher relative abundance of Pseudomonadota (*p* = 0.029) in the DS children compared with the control children. No significant differences in phylum‐level abundance were observed in the adolescents–adults group (Figure S2).

**Figure 1:**
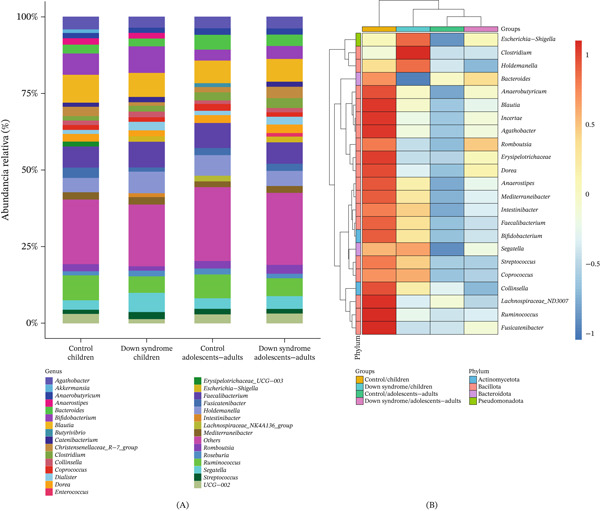
Relative abundances of bacterial taxa in study samples. (A) Bar graphs display genera with > 1% relative abundance; all others grouped as “Others.” Legend identifies genera by color. (B) Core microbiota heat map shows genera present in ≥ 20% of samples with ≥ 1% abundance. Color scale (red‐blue) indicates relative abundance; left‐side codes denote phyla. Sample sizes: DS children (*n* = 18), DS adolescents–adults (*n* = 16), control children (*n* = 27), control adolescents–adults (*n* = 11). Statistical analysis: Wilcoxon rank‐sum test for genus‐level relative abundance differences.

When analyzing relative abundance at the genus level, a higher abundance of *Ruminococcus* (*p* = 0.03) and *Fusicatenibacter* (*p* = 0.01) in the control children group, as well as the genus *Dialister* (*p* = 0.01) and *Escherichia–Shigella* (*p* = 0.04) in the DS children (Figure 1A). No differential in relative abundance was observed in the adolescents–adults groups.

A core microbiota model was constructed to explore further the microbial composition based on taxa representing > 1% of total reads and present in at least 20% of samples (Figure 1B). A heat map of normalized relative abundances highlighted distinct microbial patterns between groups. In the early‐age cohort, the control children group showed a higher relative abundance of *Bifidobacterium*, *Faecalibacterium*, *Blautia*, *Ruminococcus*, *Fusicatenibacter*, *Agathobacter*, and *Anaerobutyricum*. In contrast, the DS children group showed higher abundances of *Clostridium*, *Holdemanella*, and *Escherichia–Shigella*. In the DS adolescents–adults and control groups, the abundance of these genera generally declined, and a more similar microbial profile was observed between DS and control participants, suggesting age‐related convergence in gut microbiota composition.

### 3.3. Microbial Abundance Related to Glucose Levels

**Figure 2:**
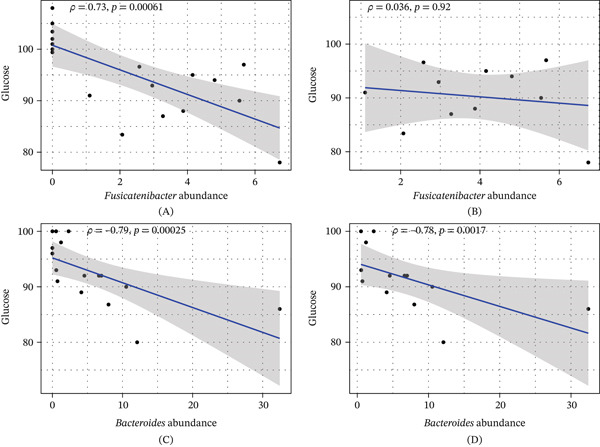
Correlation between microbial abundance and glucose. Scatterplots showing Spearman correlation coefficients between microbial abundance and glucose levels in (A) children with DS (*n* = 18) and (B) adolescents–adults with DS (*n* = 16). After filtering out zero‐abundance samples, the correlation disappears in (C) children with DS but persists in (D) adolescents–adults with DS.

To identify the possible relationship of anthropometric and biochemical parameters with bacterial abundance, due to their ability to influence metabolic functions, Spearman correlation tests were performed. In the DS children group, it was identified that the genus *Fusicatenibacter* influences glucose levels (*ρ* = −0.73, *p* < 0.001; Figure 2A). Likewise, in the DS adolescents–adults group, a negative correlation between the abundance of the *Bacteroides* genus and glucose levels was observed (*ρ* = −0.79, *p* < 0.04; Figure 2C). Although strong correlations were initially observed in both groups, sensitivity analyses revealed that these associations were no longer significant after removing zero‐abundance samples in the DS children group (Figure 2B), whereas they remained significant in the DS adolescents–adults group (Figure 2D). No correlations were established with any of the parameters in the control/children and control/adolescents–adults groups. However, results should be interpreted cautiously, as primary analyses did not adjust for diet or BMI confounders. These confounders warrant evaluation in future studies with larger cohorts. Detailed results analyses are presented in the Supporting Information (Spearman_correlation_results.xlsx).

### 3.4. Alpha and Beta Diversity of Gut Microbiota

**Figure 3:**
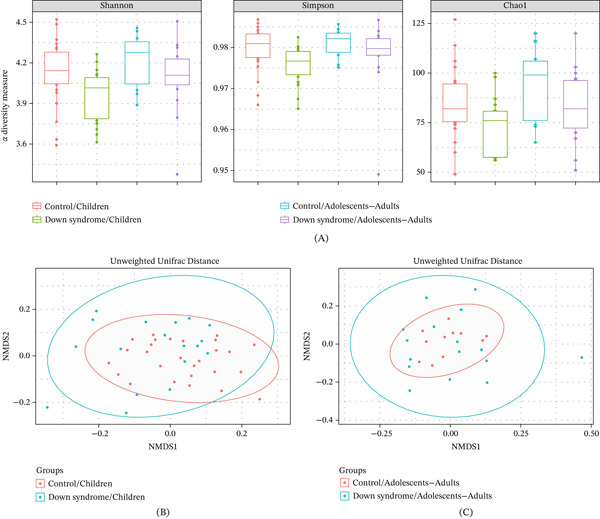
Alpha and beta diversity metrics. (A) Shannon, Simpson, and Chao1 indices compared control children (*n* = 27) with DS children (*n* = 18) using pairwise Wilcoxon rank‐sum tests (BH corrected). (B) NMDS ordination of unweighted UniFrac beta diversity for children (DS *n* = 18, control *n* = 27) and (C) adolescents–adults (DS *n* = 16, control *n* = 11), with PERMANOVA (adonis2) significance testing.

Prior to alpha diversity analyses, rarefaction curves were generated (Figure S3). These metrics revealed significantly reduced microbial diversity in DS children (Figure 3A), with lower Shannon (*p* = 0.029) and Simpson (*p* = 0.019) indices compared with controls. No differences were observed between adolescents–adults groups. Given the modest and unbalanced size of the subgroups, we performed effect size analyses (Wilcoxon *r* and *η* ^2^) to assess the biological relevance of the observed differences available in the Supporting Information (Alpha and Beta diversity.xlsx).

Beta diversity analysis revealed significant differences in microbial community structure among the children′s groups. Significant group differences were observed for unweighted UniFrac distances, indicating compositional differences driven primarily by the presence/absence of taxa. When comparing solely by group and condition (Figure 3B), a borderline significance was observed (*p* = 0.051); however, adjustment for BMI rendered these differences statistically significant (*p* = 0.017), particularly between normal‐weight control children and those with DS and overweight‐obesity (*p* = 0.03) (Figure S4).

In the adolescents–adults group (Figure 3C), significant differences emerged when comparing by condition alone (*p* = 0.024). These persisted after BMI adjustment, especially in the comparison between control adolescents–adults with normal weight and those with DS and normal weight (*p* = 0.03) (Figure S4). Detailed results and supporting analyses are presented in the Supporting Information (Alpha and Beta diversity.xlsx).

### 3.5. Differential Abundance Analysis (DESeq2) and ANCOM‐BC

**Figure 4:**
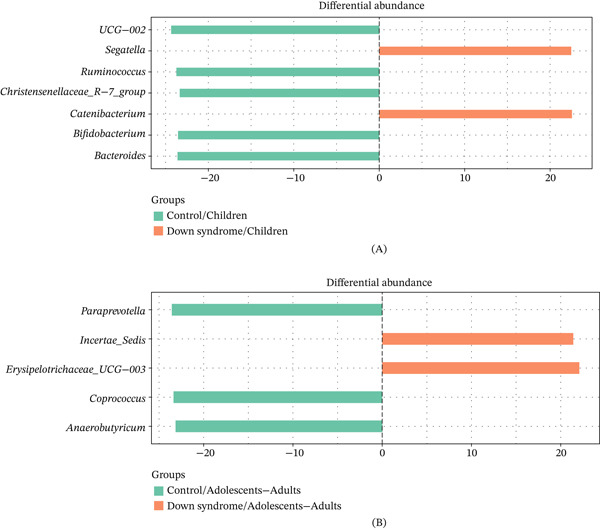
DESeq2 differential abundance analysis. Bacterial taxa with significant differential abundance between (A) DS children (*n* = 18) and control children (*n* = 27) and (B) DS adolescents–adults (*n* = 16) and control adults (*n* = 11). Analysis used the Wald test with Benjamini–Hochberg (BH) correction; *p*.adj < 0.05 taxa labeled on the *y*‐axis.

Differential abundance analysis in the DS children group identified increased abundance of *Segatella* (Bacteroidota) and *Catenibacterium* (Bacillota). Conversely, decreased abundance was noted for *Bifidobacterium, Bacteroides, Ruminococcus, and Christensenellaceae_R7_group* (Figure 4A). In the DS/Adolescent‐Adult group (Figure 4B), notable increases were seen in *Erysipelotrichaceae_UCG-003,* whereas *Paraprevotella, Coprococcus, and Anaerobutyricum* showed decreased abundance. DESeq2 detailed results are available in the Supporting Information (DESq2_results.xlsx).

The DESeq2 results were cross‐validated using ANCOM‐BC. The differentially abundant genera differed from those identified by DESeq2, highlighting only the *Sutterella* and *Enterococcus* genera in the DS children group. For the adult group, ANCOM‐BC identified no differential genera between groups. ANCOM‐BC detailed results analyses are presented in the Supporting Information (ANCOMBC_results.xlsx).

### 3.6. Functional Enrichment Analysis of Bacterial Metagenomes in Children and Adolescents–Adults

Functional profiling of the bacterial metagenome, predicted using PICRUSt2 and analyzed through KEGG pathway enrichment, revealed highly similar core functional profiles across both age groups‐children and adolescents–adults, and between control and DS cohorts (Figure S5). The most significantly enriched pathways in all groups included the biosynthesis of cofactors, amino acids, and nucleotide sugars, as well as the metabolism of fructose and mannose, pyruvate, and glycolysis/gluconeogenesis. These pathways, fundamental to bacterial metabolism and cellular functions, were consistently represented with high gene ratios and strong statistical significance.

Enrichment analysis reveals over 90% compatibility in predicted metagenomic functionality. However, these results warrant cautious interpretation, as they do not reflect the microbiota′s actual functional activity. Additional details and results are provided in the Supporting Information (KEGG_enrichment.xlsx).

## 4. Discussion

Individuals with DS are vulnerable to a range of phenotypes related to metabolic and gastrointestinal disorders, such as obesity, diabetes, and constipation [1, 4], and neurological disorders such as anxiety, depression, and Alzheimer′s disease [3, 5]. Our study confirms that growth impairment in DS manifests early in life, consistent with previous research showing growth delays during developmental stages. These impairments have been attributed to dysfunctions within the growth hormone release hormone—growth hormone—insulin‐like growth factor 1 axis [29, 30]. Moreover, hormonal therapies have shown promise in restoring more typical growth trajectories [31].

Although DS was once considered an atheroma‐free model, recent studies indicate an increased risk of mortality from ischemic heart disease. Furthermore, atherosclerotic lesions have been observed in coronary arteries obtained from autopsies of young individuals with DS. Specifically, we observed lower HDL‐c levels in the DS children group compared with controls, even after adjusting for BMI. This supports existing evidence of an abnormal lipid profile and increased cardiovascular risk in DS, emphasizing the influence of genetic conditions on lipid metabolism [32, 33]. A recent meta‐analysis also underscores the importance of monitoring lipid profiles in this population [34].

The gut microbiota plays a crucial role in host health; however, its composition is highly susceptible to being disrupted by factors such as lifestyle, antibiotics, mode of delivery, and genetics [10]. Early‐life alterations in gut microbiota have been associated with long‐term metabolic [22], immune [23], and neurological disorders [24]. Characterizing gut microbiota is particularly important in people with DS, who have an increased risk of metabolic and functional bowel disorders [1, 4], as well as neurological disorders [3, 5].

Previous studies have linked alterations in alpha diversity metrics, such as decreased Shannon and increased Simpson indices, to disorders like autism, obesity, diabetes, and Alzheimer′s disease [35–38]. In our study, we found a significant decrease in the Simpson index (*p* < 0.019) and the Shannon index (*p* < 0.029) in the DS children group compared with the controls. These differences were not observed in the adolescents–adults group. Our findings are consistent with studies reporting alterations in the microbiota in children with DS, particularly regarding cognitive function [14]. However, other researchers have reported inconsistent alpha diversity results, possibly due to differences in study populations, findings that align with our observations in the adolescents–adults group [7, 13]. To our knowledge, this is the first study to perform age stratification in this context.

Beta diversity also differed significantly between the children in the DS and control groups affected by BMI, consistent with previous findings [14]. Early‐stage dysbiosis may be associated with chronic alterations in individuals with DS. This critical window of microbial development can influence metabolic, physiological, immune, and neurological pathways [11, 39, 40]. Interestingly, such alterations have been suggested to be reversible during early life, highlighting the need for early intervention strategies to promote a healthy microbiome [12].

At the phylum level, we found an increased abundance of Pseudomonadota (*p* < 0.029) in the DS children group. These differences were not present in the adolescents–adults group. Pseudomonadota are associated with gut inflammation and may contribute to metabolic diseases by producing lipopolysaccharide (LPS), which can compromise the intestinal barrier and trigger low‐grade inflammation [41–43].

Differential abundance analysis at the genus level revealed increased *Segatella* and *Catenibacterium* in the DS children group. *Prevotella* has been linked to increased levels of proinflammatory cytokines and behavioral abnormalities in animal models [7, 44, 45]. Its role in the gut–brain axis, a complex bidirectional network, is significant, and LPS‐induced inflammation may lead to cognitive decline [46, 47]. Additionally, *Prevotella copri* is associated with insulin resistance and cardiovascular risk [48]. The proinflammatory environment in DS may further support colonization of *Prevotella* due to the presence of superoxide reductase [49, 50].

*Catenibacterium* has also been associated with obesity‐related biomarkers and increased proinflammatory cytokines [7, 51]. In contrast, beneficial genera such as *Bifidobacterium*, *Bacteroides,* and *Ruminococcus* were more abundant in controls. These genera support intestinal barrier integrity, immune modulation, pathogen inhibition, and cognitive function through the production of SCFAs and neurotransmitters such as gamma‐aminobutyric acid (GABA) [52–54].

Interestingly, deficits in GABAergic signaling have been observed in individuals with DS. This dysfunction is partly caused by increased expression of Na^+^, K^+,^ and Cl^−^ cotransporter 1 (NKCC1), which disrupts the balance between excitatory and inhibitory neural signals. Such an imbalance is believed to contribute to impaired brain development, cognitive deficits, and possibly behavioral disorders [55, 56]. However, the mechanisms behind NKCC1 overexpression remain unclear. Recent studies suggest that a chronic proinflammatory state can alter neurotransmitter function [57]. Notably, tumor necrosis factor (TNF‐*α*), a cytokine consistently elevated in the DS population, has been identified as a positive regulator of NKCC1 expression [7, 58]. This proinflammatory condition may be intensified by intestinal dysbiosis, a hallmark of DS, leading to increased production of proinflammatory cytokines, higher intestinal permeability, LPS endotoxemia, and systemic immune activation. These changes weaken the blood–brain barrier and promote neuroinflammation, ultimately impacting neurotransmitter activity [7, 59].

The role of gut microbiota in modulating brain function has gained increasing attention. In particular, *Bifidobacterium* species have demonstrated beneficial effects on mood and behavioral disorders by influencing the gut–brain axis, in which the vagus nerve plays a central role [60–62]. These effects are attributed to *Bifidobacterium*′s ability to modulate neurotransmitter systems, including increasing GABA levels, promoting serotonin (5‐HT) production via tryptophan metabolism, and reducing systemic inflammation [63, 64]. These findings highlight the potential of psychobiotics like *Bifidobacterium* as therapeutic agents targeting neuropsychiatric and cognitive systems in DS.

Our results should be interpreted as exploratory because they could not be reproduced through cross‐validation. On the other hand, *Sutterella* and *Enterococcus* were identified as differential genera using ANCOM‐BC. The genus *Sutterella* has been detected in DS populations and is associated with cognitive impairment [13]. It also appears enriched in individuals with autism spectrum disorder, gastrointestinal disorders, and metabolic syndrome [65–67]. *Sutterella* species (Betaproteobacteria family) are Gram‐negative, nonspore‐forming bacilli that thrive in microaerophilic or anaerobic conditions; they can produce LPS, promoting intestinal permeability and inflammation. However, recent research indicates that *Sutterella* spp. lack sufficient enzymes for LPS lipid A synthesis, a key component of the inflammatory response, suggesting immunomodulatory functions [68]. Further studies are needed to elucidate the mechanisms linking *Sutterella* to metabolic and cognitive disorders.

Conversely, the *Enterococcus* genus is associated with various beneficial effects. However, in cases of intestinal dysbiosis, species such as *E. faecalis* and *E. faecium* can become pathogenic when they proliferate excessively, translocate across the intestinal barrier, and contribute to clinical disease [69, 70]. The presence of antimicrobial resistance genes, combined with the production of virulence factors and bile acid modification, enhances their pathogenic potential and abundance [70, 71].

Emerging evidence supports the effectiveness of early pharmacological interventions in modulating clinical phenotypes in DS, particularly when applied during critical developmental periods [72, 73]. In addition, microbiota and nutrition‐based therapies show promise as complementary strategies with possibly age‐dependent effects. These findings highlight the importance of establishing a healthy gut microbiota early in life to promote optimal neurodevelopment and long‐term health [12, 23, 74–77].

Nonetheless, to date, no microbiota‐based therapeutic strategies have been developed and tested in DS, except for a recent report documenting the use of symbiotics in a mouse model of DS [78]. Such a background provides an initial foundation but also offers a promising opportunity for future research to identify early interventions to restore gut microbial balance and mitigate phenotypic variability in this vulnerable population.

Functional metagenomic analysis revealed a high concordance in predicted functional profiles between different groups. Despite changes in microbiota composition, these were not reflected in functional differences, likely due to functional redundancy within the microbial community. To assess actual microbial functionality, approaches such as metabolomics, which directly capture the metabolic output of the microbial ecosystem, are therefore essential. Therefore, these findings should be interpreted with caution, as they reflect predicted rather than actual functional capacity.

The limitations of this study should be considered. Our study is cross‐sectional, which prevents the assessment of causal relationships; longitudinal studies are needed to track changes in the microbiota over time and their association with clinical outcomes. Some factors, such as diet, are necessary to evaluate microbiota composition. The study primarily reports microbiota composition and some clinical parameters but does not deeply explore the correlation between specific microbial changes and clinical phenotypes, such as cognitive function. Future studies are needed to evaluate these features.

## 5. Conclusion

The present study provides evidence of reduced serum HDL cholesterol levels in a sample of individuals affected with DS during early life, suggesting an increased risk for future cardiovascular complications. Additionally, we observed gut microbiota dysbiosis in the DS population, with more pronounced alterations at younger ages. These early‐life disruptions in microbial composition may be associated with impaired neurodevelopment, altered metabolic regulation, and chronic low‐grade inflammation. Our findings underscore the importance of early monitoring and potential intervention strategies to restore microbial balance. Further studies with larger cohorts are needed to validate these findings and to elucidate the underlying mechanism linking gut dysbiosis with systemic and neurological outcomes in DS.

## Author Contributions

Jaime García‐Mena and Javier Magaña Gómez contributed equally to this project.

## Funding

No funding was received for this manuscript.

## Conflicts of Interest

The authors declare no conflicts of interest.

## Supporting Information

Additional supporting information can be found online in the Supporting Information section.

## Supporting information


**Supporting Information 1** Figure S1: Flow diagram of participant recruitment and selection process for the DS microbiome study. The study began with 98 DS cases and 110 controls (total *n* = 208). Participants provided written informed consent and completed medical history questionnaires. Exclusion criteria included antibiotic use within the previous 6 months, probiotics/prebiotics consumption, specific dietary regimens, and failure to provide consent or samples. After exclusions (64 DS cases and 72 controls), the final analytical cohort comprised 34 DS cases and 38 controls (total *n* = 72). Participants were stratified by age into children (≤ 12 years: 18 DS, 27 controls) and adolescents–adults (> 12 years: 16 DS, 11 controls) for comparative analyses.

**Supporting Information 2** Figure S2: Relative abundance of bacterial phyla. Bar chart showing phyla‐level composition across study groups; color‐coded segments represent phyla (legend right). *y*‐axis: relative abundance percentages (< 1% phyla grouped as “Others”). Sample sizes: DS children (*n* = 18), DS adolescents–adults (*n* = 16), control children (*n* = 27), and CONTROL adolescents–adults (*n* = 11). Bacillota predominates, followed by Bacteroidota, Actinomycetota, Pseudomonadota, and Verrucomicrobiota. Statistical analysis: Wilcoxon rank‐sum test for intergroup differences.

**Supporting Information 3** Figure S3: Rarefaction curve. Sequencing depth standardized to 5818 reads per sample, where curves plateaued, confirming adequate sampling for diversity analyses. Sample sizes: DS children (*n* = 18), DS adolescents–adults (*n* = 16), control children (*n* = 27), and control adolescents–adults (*n* = 11); total *n* = 72.

**Supporting Information 4** Figure S4: Beta diversity structure by BMI. Nonmetric multidimensional scaling (NMDS) plots. Nonmetric multidimensional scaling (NMDS) plots based on unweighted UniFrac distances illustrate microbial community dissimilarities across BMI categories: (A) children and (B) adolescents–adults. PERMANOVA (adonis2) confirmed significant differences (*p* < 0.05) after BMI adjustment.

**Supporting Information 5** Figure S5: KEGG pathway enrichment analysis of metagenome predictions by PICRUSt2. Panels show statistically significant pathways for (A) control children, (B) DS children, (C) control adolescents–adults, and (D) DS adolescents–adults. Sample sizes: DS children (*n* = 18), DS adolescents–adults (*n* = 16), control children (*n* = 27), and control adolescents–adults (*n* = 11). Red/blue bars indicate higher/lower enrichment (*P* values); *x*‐axis: gene ratio; *y*‐axis: pathways (ordered by significance). Statistical analysis: Fisher′s exact test with Benjamini–Hochberg correction.

**Supporting Information 6** File S1: Alpha and beta diversity. Supporting information containing alpha and beta diversity analyses of the gut microbiota across control and Down syndrome groups, stratified by age and nutritional status, including Wilcoxon tests, effect size estimates, NMDS, and PERMANOVA.

**Supporting Information 7** File S2: Spearman′s correlation results. The supporting information presents correlations between taxon abundance and biochemical and anthropometric variables in children and adults with Down syndrome and their respective controls.

**Supporting Information 8** File S3: DESq2 results. The supporting information presents the differential taxon analysis results between the study groups.

**Supporting Information 9** File S4: ANCOMBC results. The supporting information presents the differential taxon analysis results between the study groups.

**Supporting Information 10** File S5: KEGG enrichment. The supporting information presents the results of the statistical analysis of enriched pathways using the KEGG database.

## Data Availability

The data that support the findings of this study are available on request from the corresponding authors. The data are not publicly available due to privacy or ethical restrictions.
