# Supplementary material for: Gut Microbiota Differences in Down Syndrome Are Most Pronounced in Childhood and Diminish With Age
Source: Int J Microbiol. 2026 Jul 9;2026:6617119. doi: 10.1155/ijm/6617119 (PMC13351334; doi:10.1155/ijm/6617119)
Supplement: Supplementary file 1 — Supporting Information 1 Figure S1: Flow diagram of participant recruitment and selection process for the DS microbiome study. The study began with 98 DS cases and 110 controls (total n = 208). Participants provided written informed consent and completed medical history questionnaires. Exclusion criteria included antibiotic use within the previous 6 months, probiotics/prebiotics consumption, specific dietary regimens, and failure to provide consent or samples. After exclusions (64 DS cases and 72 controls), the final analytical cohort comprised 34 DS cases and 38 controls (total n = 72). Participants were stratified by age into children (≤ 12 years: 18 DS, 27 controls) and adolescents–adults (> 12 years: 16 DS, 11 controls) for comparative analyses. [file IJM-2026-6617119-s002.zip › 6617119.f1.xml]

Initial samples:

98 DS

110 Controls

Written informed consent

Sample collection

Medical history

Exclusion criteria

- Antibiotics used
- Especific diets
- Probiotics/prebiotics used
- Failure to provide samples
- Failure to sign consent form

Excluded:

64 cases DS

72 Controls

Adolescents-Adults (>12 years)
16 cases DS

11 Controls

Children (4-12 years)
18 cases DS

27 Controls

Age stratification

Final sample:
34 cases DS

38 Controls

**Figure S1. Flow diagram of participant recruitment and selection process for the Down syndrome (DS) microbiome study.** The study began with 98 DS cases and 110 controls (total n=208). Participants provided written informed consent and completed medical history questionnaires. Exclusion criteria included antibiotic use within the previous 6 months, probiotics/prebiotics consumption, specific dietary regimens, and failure to provide consent or samples. After exclusions (64 DS cases and 72 controls), the final analytical cohort comprised 34 DS cases and 38 controls (total n=72). Participants were stratified by age into children (≤12 years: 18 DS, 27 controls) and adolescents-adults (>12 years: 16 DS, 11 controls) for comparative analyses.​
