# Supplementary figures and images for: Gut Microbiota Differences in Down Syndrome Are Most Pronounced in Childhood and Diminish With Age
Source: Int J Microbiol. 2026 Jul 9;2026:6617119. doi: 10.1155/ijm/6617119 (PMC13351334; doi:10.1155/ijm/6617119)

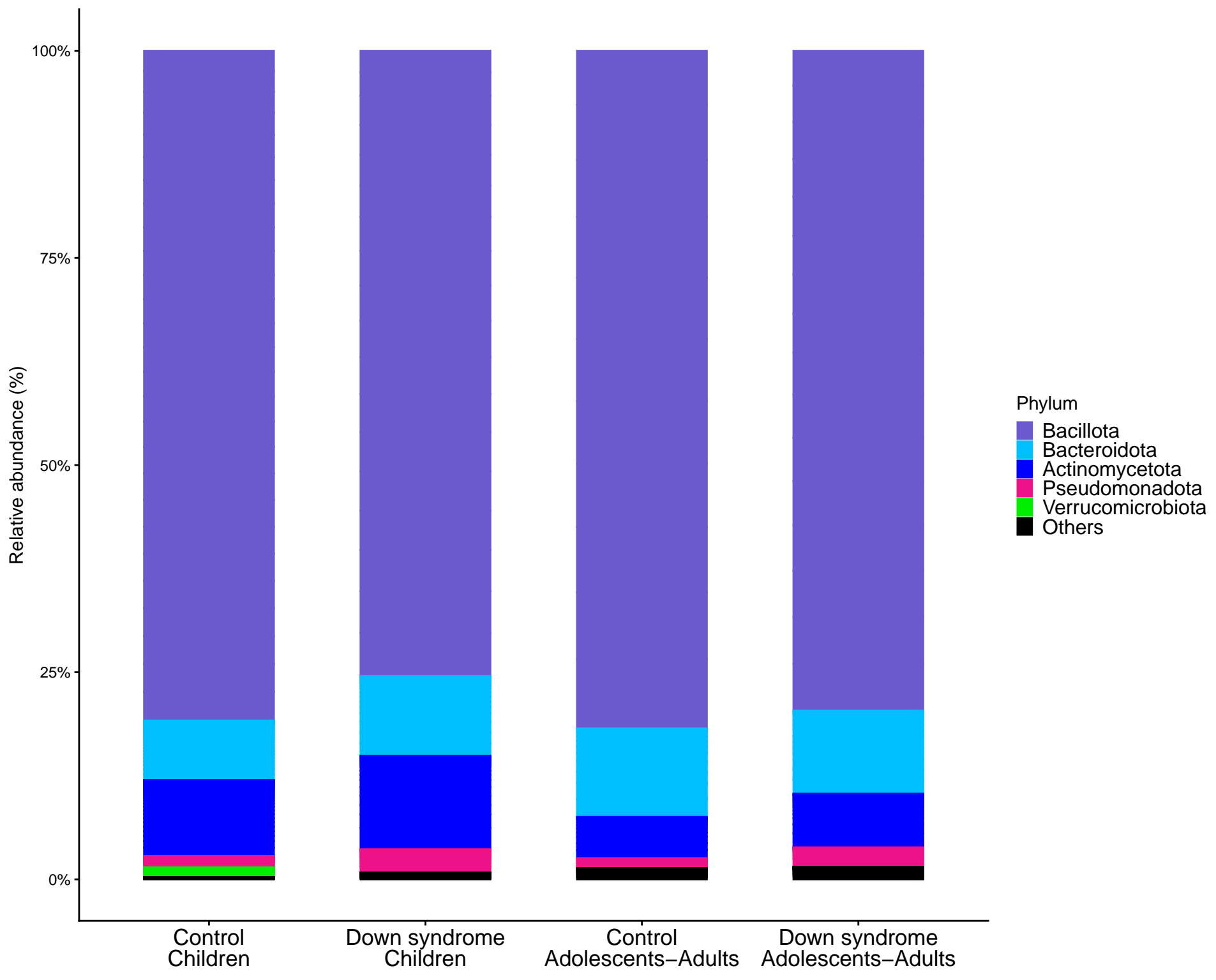

Supplement: Supplementary file 2 — Supporting Information 2 Figure S2: Relative abundance of bacterial phyla. Bar chart showing phyla‐level composition across study groups; color‐coded segments represent phyla (legend right). y‐axis: relative abundance percentages (< 1% phyla grouped as “Others”). Sample sizes: DS children (n = 18), DS adolescents–adults (n = 16), control children (n = 27), and CONTROL adolescents–adults (n = 11). Bacillota predominates, followed by Bacteroidota, Actinomycetota, Pseudomonadota, and Verrucomicrobiota. Statistical analysis: Wilcoxon rank‐sum test for intergroup differences. [file IJM-2026-6617119-s005.pdf]

**A**

Unweighted Unifrac Distance

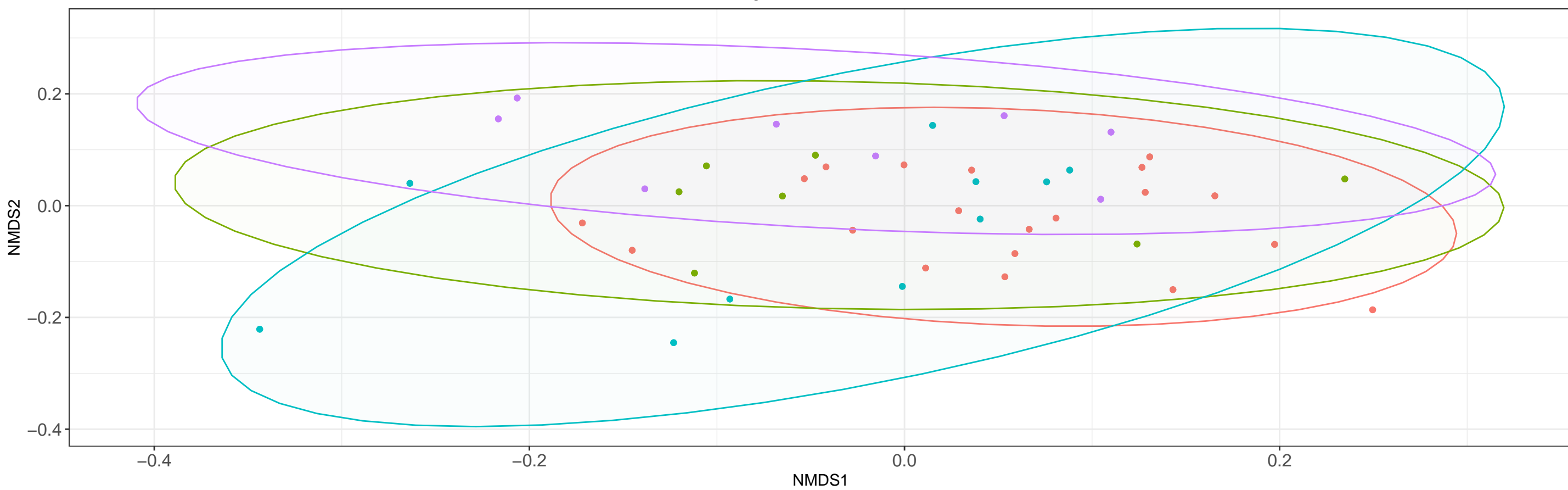**B**

Unweighted Unifrac Distance

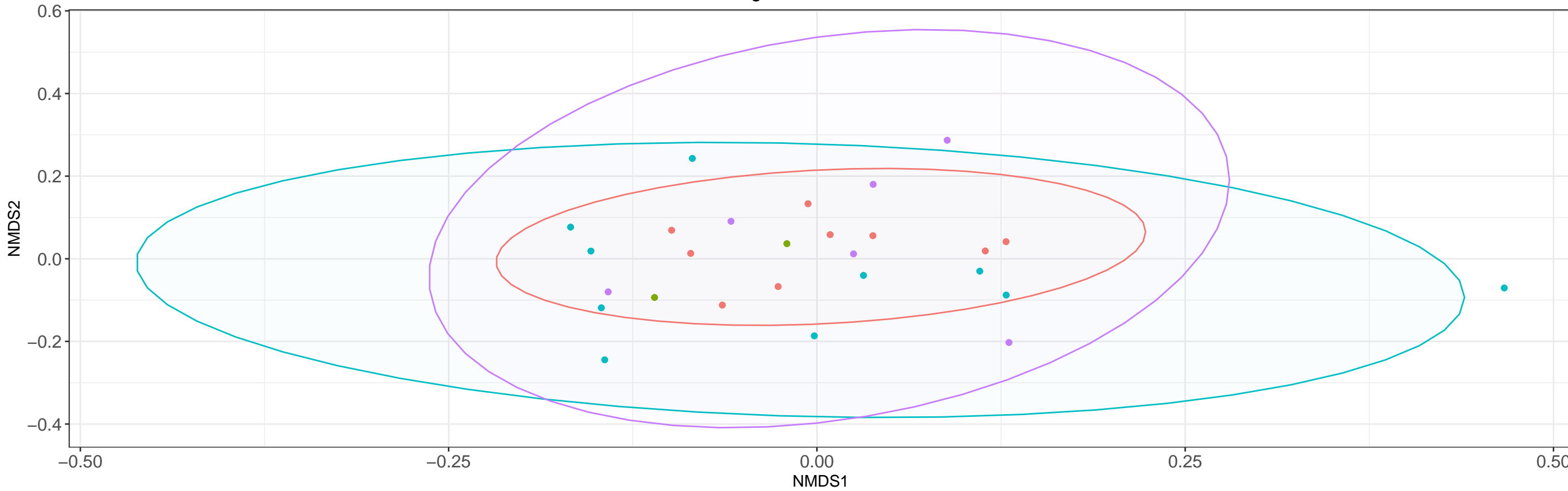

Supplement: Supplementary file 4 — Supporting Information 4 Figure S4: Beta diversity structure by BMI. Nonmetric multidimensional scaling (NMDS) plots. Nonmetric multidimensional scaling (NMDS) plots based on unweighted UniFrac distances illustrate microbial community dissimilarities across BMI categories: (A) children and (B) adolescents–adults. PERMANOVA (adonis2) confirmed significant differences (p < 0.05) after BMI adjustment. [file IJM-2026-6617119-s003.pdf]

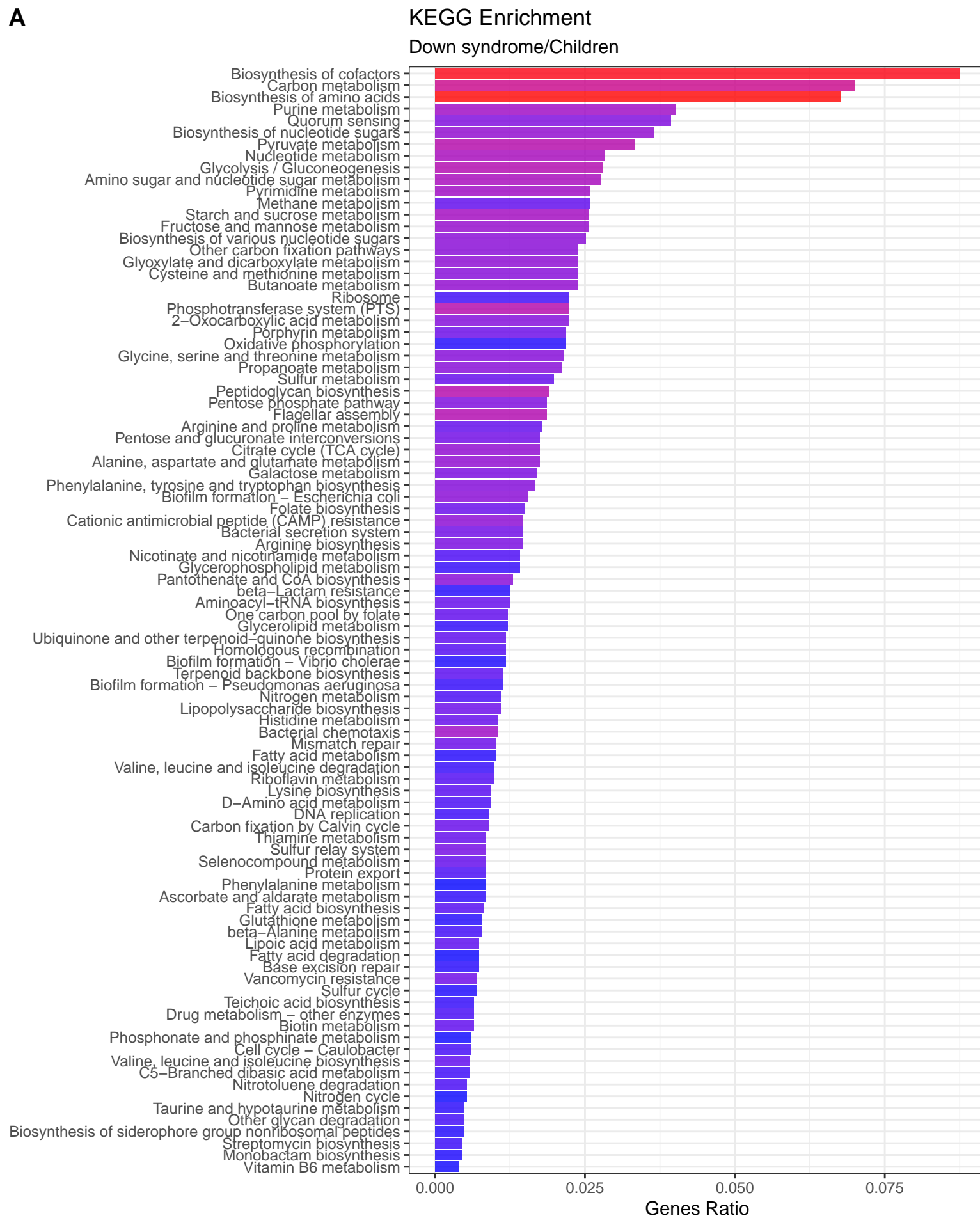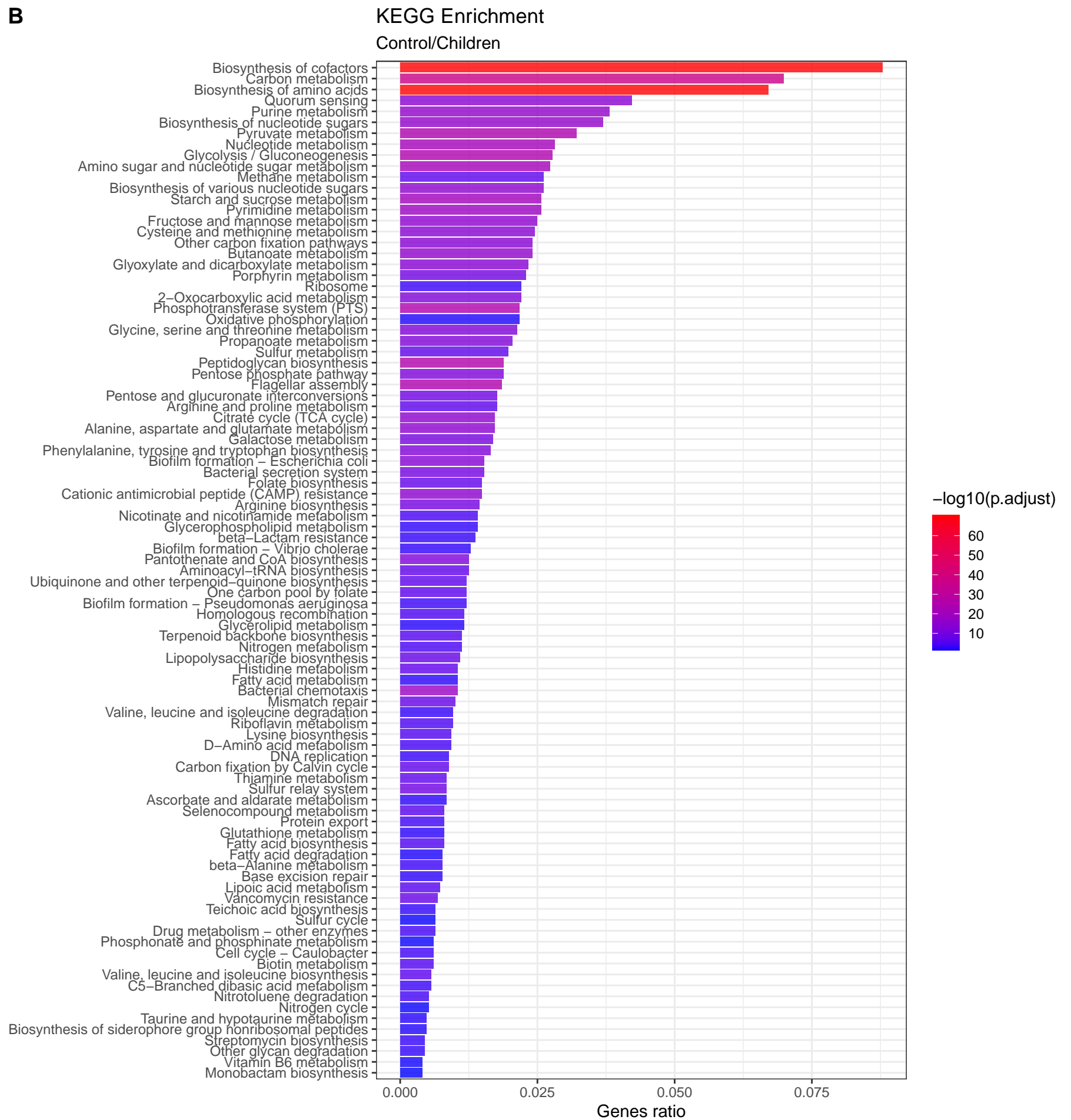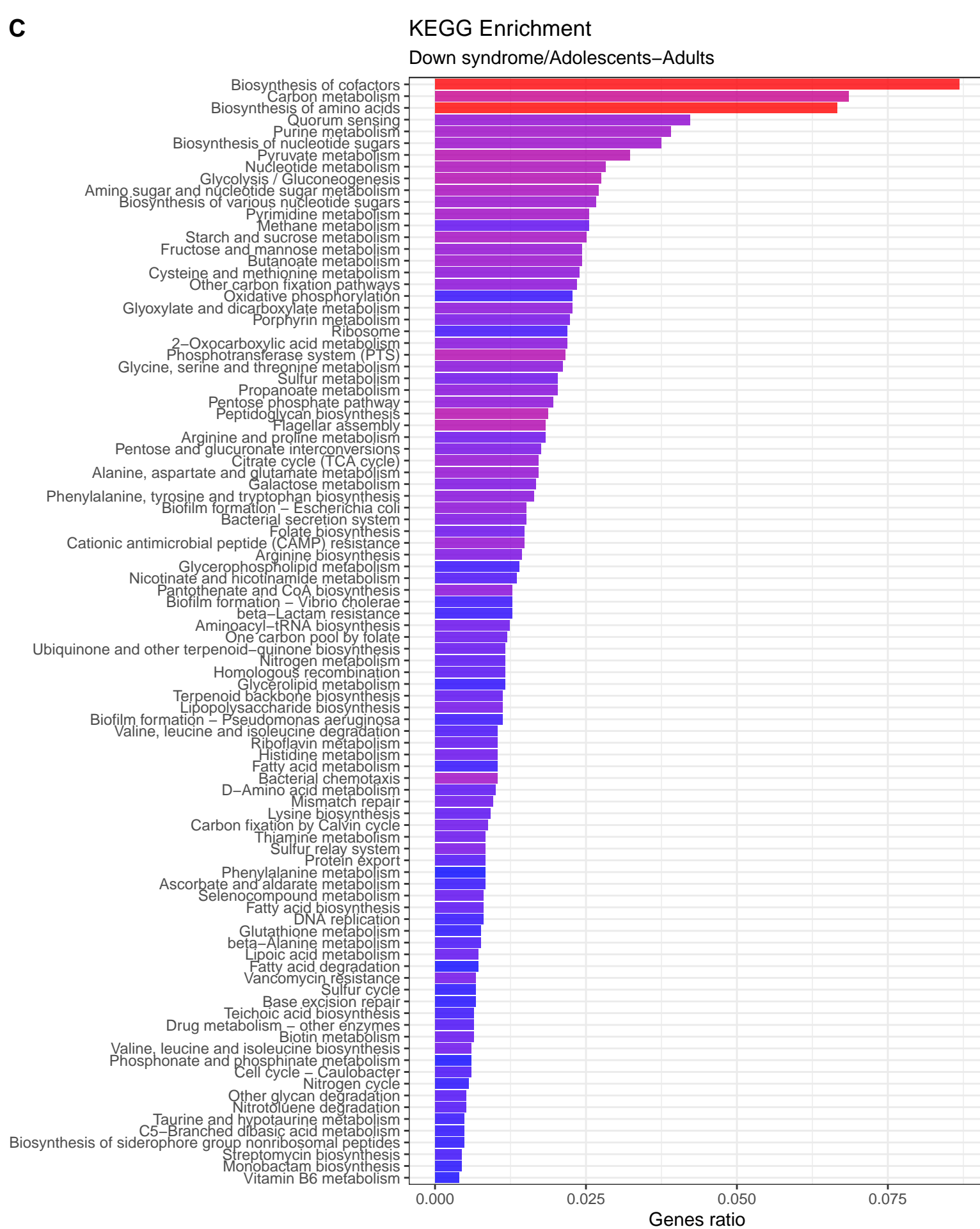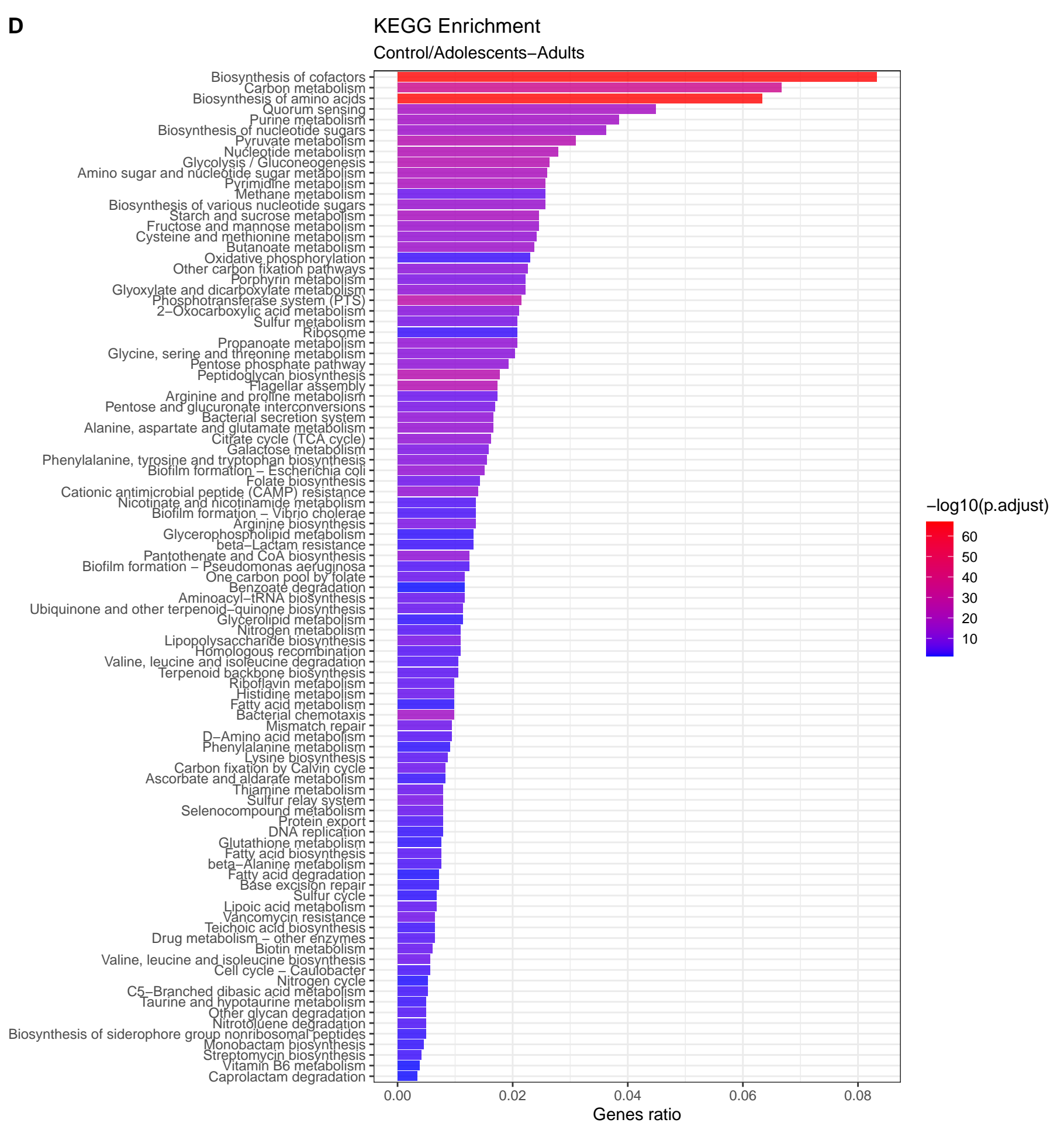

Supplement: Supplementary file 5 — Supporting Information 5 Figure S5: KEGG pathway enrichment analysis of metagenome predictions by PICRUSt2. Panels show statistically significant pathways for (A) control children, (B) DS children, (C) control adolescents–adults, and (D) DS adolescents–adults. Sample sizes: DS children (n = 18), DS adolescents–adults (n = 16), control children (n = 27), and control adolescents–adults (n = 11). Red/blue bars indicate higher/lower enrichment (P values); x‐axis: gene ratio; y‐axis: pathways (ordered by significance). Statistical analysis: Fisher′s exact test with Benjamini–Hochberg correction. [file IJM-2026-6617119-s006.pdf]
