# Supplementary material for: Gut Microbiota Differences in Down Syndrome Are Most Pronounced in Childhood and Diminish With Age
Source: Int J Microbiol. 2026 Jul 9;2026:6617119. doi: 10.1155/ijm/6617119 (PMC13351334; doi:10.1155/ijm/6617119)
Supplement: Supplementary file 3 — Supporting Information 3 Figure S3: Rarefaction curve. Sequencing depth standardized to 5818 reads per sample, where curves plateaued, confirming adequate sampling for diversity analyses. Sample sizes: DS children (n = 18), DS adolescents–adults (n = 16), control children (n = 27), and control adolescents–adults (n = 11); total n = 72. [file IJM-2026-6617119-s001.pdf]

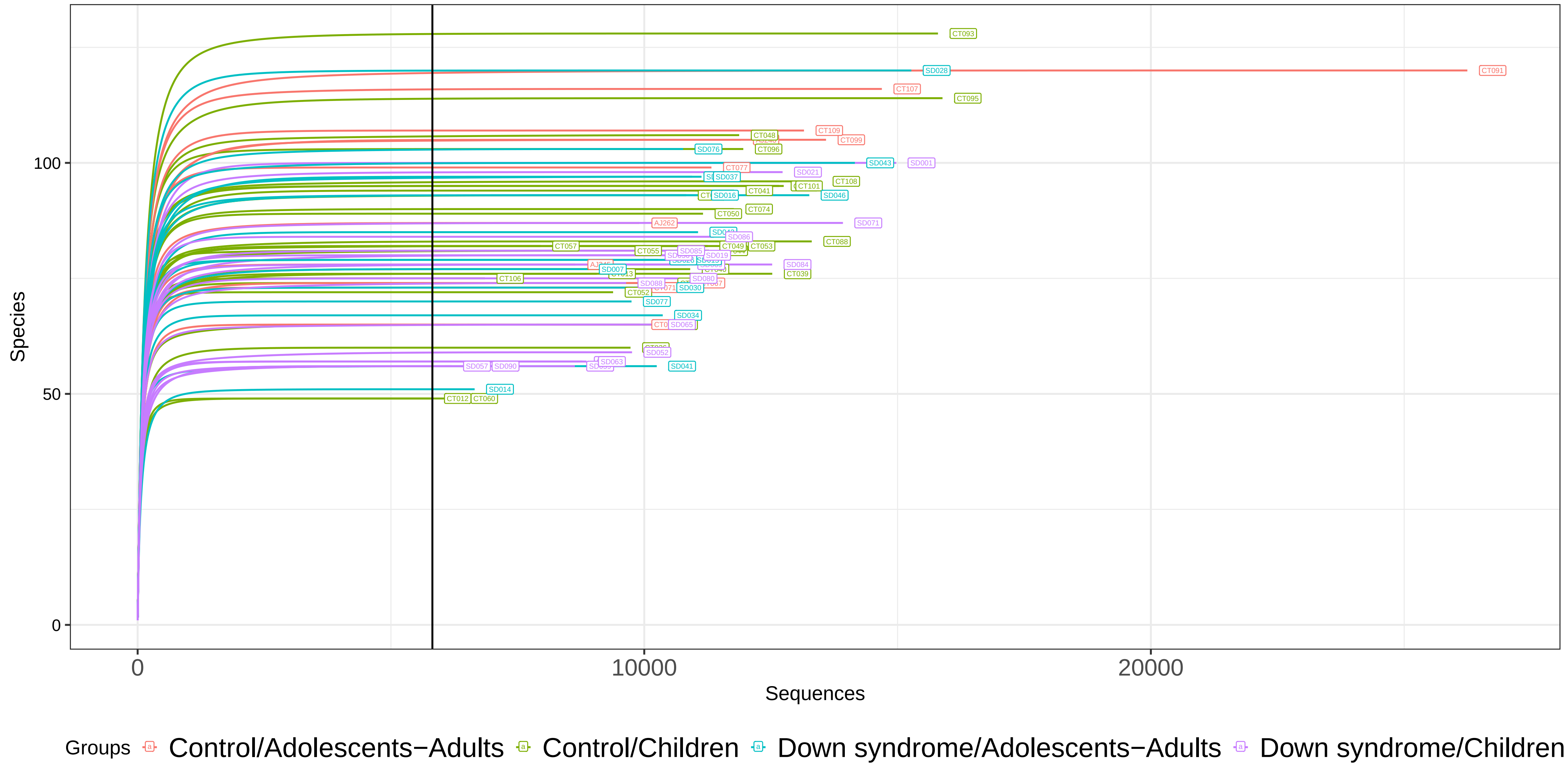

Species
Sequences
0
50
100
0
10000
20000
Groups
Control/Adolescents−Adults
Control/Children
Down syndrome/Adolescents−Adults
Down syndrome/Children
CT093
SD028
CT091
CT107
CT095
CT109
CT048
CT099
SD076
CT096
SD043
SD001
CT077
SD021
SD037
CT108
CT101
CT041
SD016
SD046
CT074
CT050
AJ262
SD071
SD086
CT088
CT057
CT049
CT053
CT055
SD085
SD019
SD084
SD007
CT039
CT106
SD080
SD088
SD030
CT052
SD077
SD034
SD065
SD052
SD063
SD057
SD090
SD041
SD014
CT012
CT060
